# Microbial Type IA Topoisomerase C-Terminal Domain Sequence Motifs, Distribution and Combination

**DOI:** 10.3390/ijms23158709

**Published:** 2022-08-05

**Authors:** Brenda Diaz, Christopher Mederos, Kemin Tan, Yuk-Ching Tse-Dinh

**Affiliations:** 1 Department of Chemistry and Biochemistry, Florida International University, Miami, FL 33199, USA; 2 Structural Biology Center, X-ray Science Division, Advanced Photon Source, Argonne National Laboratory, 9700 S. Cass Avenue, Lemont, IL 60439, USA; 3 Biomolecular Sciences Institute, Florida International University, Miami, FL 33199, USA

**Keywords:** topoisomerase, type IA, TopA, Top3, zinc fingers, zf-GRF, zf-CCHC

## Abstract

Type IA topoisomerases have highly conserved catalytic N-terminal domains for the cleaving and rejoining of a single DNA/RNA strand that have been extensively characterized. In contrast, the C-terminal region has been less covered. Two major types of small tandem C-terminal domains, Topo_C_ZnRpt (containing C4 zinc finger) and Topo_C_Rpt (without cysteines) were initially identified in *Escherichia coli* and *Mycobacterium tuberculosis* topoisomerase I, respectively. Their structures and interaction with DNA oligonucleotides have been revealed in structural studies. Here, we first present the diverse distribution and combinations of these two structural elements in various bacterial topoisomerase I (TopA). Previously, zinc fingers have not been seen in type IA topoisomerases from well-studied fungal species within the phylum Ascomycota. In our extended studies of C-terminal DNA-binding domains, the presence of zf-GRF and zf-CCHC types of zinc fingers in topoisomerase III (Top3) from fungi species in many phyla other than Ascomycota has drawn our attention. We secondly analyze the distribution and combination of these fungal zf-GRF- and zf-CCHC-containing domains. Their potential structures and DNA-binding mechanism are evaluated. The highly diverse arrangements and combinations of these DNA/RNA-binding domains in microbial type IA topoisomerase C-terminal regions have important implications for their interactions with nucleic acids and protein partners as part of their physiological functions.

## 1. Introduction

Type IA topoisomerases are present in all kingdoms of life to solve topological problems encountered in vital cellular processes including replication, transcription, recombination, and repair that require passing of DNA across a single DNA strand [1,2,3]. This is also the only subclass of topoisomerases that can act as RNA topoisomerases [4]. Type IA topoisomerases are characterized with highly conserved catalytic N-terminal domains (D1-D4) that assemble into a torus-like shape that have been observed in a number of crystal structures [5,6,7,8,9,10,11,12]. In contrast, the structure of the C-terminal region that follows the toroidal assembly has been much less explored. The presence of small, presumably DNA-binding domains in tandem has shown structural and functional diversity in the C-terminal region of topoisomerase IA [13]. Two major types of C-terminal domains, Topo_C_ZnRpt (containing C4 zinc finger) and Topo_C_Rpt (without cysteine), were initially identified in *Escherichia coli* topoisomerase I (EcTOP1) [14,15,16,17] and *Mycobacterium tuberculosis* topoisomerase I (MtbTOP1) [8], respectively, based on their sequence similarities, including the presence/absence of a zinc finger motif. Besides these structural domains, certain extended, positively charged sequence motifs frequently appear in the topoisomerase IA C-terminal region, sometimes as an insertion within a domain, or as a linker between two domains, or simply as the only C-terminal element by itself [18,19,20]. Structural investigation of the topoisomerase IA C-terminal region has been largely hindered by the difficulty in crystallization of these small tandem C-terminal domains due to the flexibility between domains, and the complexity introduced by the presence of long, positively charged sequence motifs. However, from co-crystallization with oligonucleotides of varied lengths, the structures of representative Topo_C_ZnRpt domains (D5-D7 of EcTOP1) and Topo_C_Rpt domains (D5-D8 of MtbTOP1 and *Mycobacterium smegmatis* topoisomerase I (MsmTOP1)) have been determined [8,17,21] and their unique DNA-binding properties characterized [15,21]. The Topo_C_ZnRpt domain is also called the zinc ribbon domain in related literature [16,17,22,23].

Besides the prototypical arrangement of these two major types of C-terminal domains in EcTOP1 and MtbTOP1/MsmTOP1, respectively, in this paper, we will present the extensive variation in the distribution and combination of these two types of C-terminal domains in topoisomerase I (encoded by the *topA* gene) from different bacterial species. This is followed by the exploration of other types of C-terminal domain repeats that are much less studied but commonly present in type IA topoisomerases identified in many fungal species. The great species richness in the fungal kingdom is of immense significance as fungi can cause widespread diseases in human, animals, and plants, as well as offer great promise for their application in pharmaceutical and industrial biotechnology. Similar to topoisomerase III in *E. coli* (encoded by *topB* gene), the type IA topoisomerase III that have been characterized for the most commonly studied fungal species such as *Saccharomyces cerevisiae* and *Schizosaccharomyces pombe* are known to have short C-terminal sequences that do not have repetitive elements. Unexpectedly, we noticed that repeats of zf-GRF and zf-CCHC zinc fingers exist in the C-terminal region of topoisomerase III (Top3) from many fungal species outside the most commonly studied phylum Ascomycota. The feature of their distribution in fungal topoisomerase III is analyzed. The possible structures and models for DNA/RNA binding of these zinc fingers found in a representative topoisomerase III of *Puccinia graminis f.* sp. *tritici* (Uniprotein A0A5B0PD53) are predicted. Comparisons are made between these new types of C-terminal domains with the bacterial topoisomerase I C-terminal repeats. The expanded knowledge of the microbial type IA topoisomerase C-terminal domains found in bacteria and fungi indicate that they could potentially engage diverse nucleic acid substrates as well as protein interaction partners for their individual specific physiological functions.

## 2. Results

### 2.1. Topo_C_ZnRpt and Topo_C_Rpt in Bacterial Topoisomerase I C-terminal Domains

#### 2.1.1. Distribution

**Figure 1:**
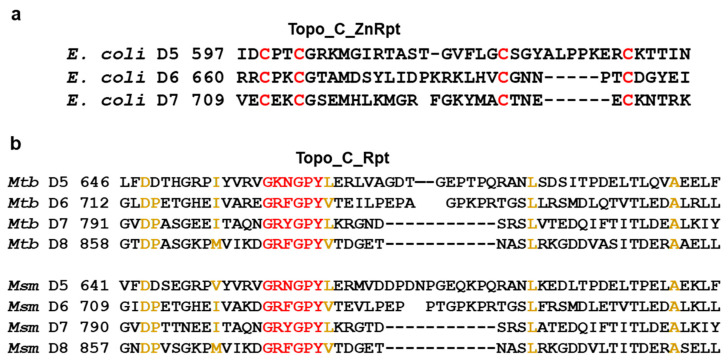
Prototype sequences for bacterial topoisomerase I C-terminal domains. (**a**) Topo_C_ZnRpt in EcTOP1. Cysteines for Zn^2+^ coordination are colored in red. (**b**) Topo_C_Rpt in MtbTOP1 and MsmTOP1. Residues that are part of the signature sequence are colored in red. Other highly conserved residues are colored in gold.

Repeats that utilize four cysteines for Zn^2+^ coordination were first identified in the C-terminal region of EcTOP1 (Figure 1a) [14]. It was later noted that the amino acid sequences of mycobacterial topoisomerase I do not have similar zinc finger motifs [24]. A suggestion was then made that the loss of zinc fingers from the topoisomerase I in Actinobacteria including Mycobacterium species could be associated with Zn^2+^ export and homeostasis [18]. Paucity of the Zn^2+^ ions may have resulted from the enhancement of Zn^2+^ export mechanisms in these organisms to avoid Zn^2+^ toxicity. It is also possible that the loss of zinc fingers with cysteines would enhance resistance to change in pH or oxidative stress [18]. The Topo_C_Rpt was subsequently identified as a repeated motif for DNA binding in the C-terminal region of MtbTOP1/MsmTOP1 sequences and structures (Figure 1b). Interestingly, the Pfam entry for Topo_C_Rpt, referred to as Toprim_C_rpt (PF13368) in the Pfam database lists 4060 species of bacteria that have this type of structural domain in topoisomerase I found mainly in the phyla of Actinobacteria, Bacteroidetes, and Proteobacteria. As shown in Table 1, the number of species from phylum Proteobacteria is about the same as the number of species from Actinobacteria. The Sunburst illustration of the species (Figure S1) shows 999 species in the Actinomycetia class from Actinobacteria and 860 species of the Alphaproteobacteria class from Proteobacteria. Therefore, the Topo_C_Rpt structural motif without Zn-binding cysteines observed initially in mycobacteria are not limited to Actinobacteria (Table 1). Interestingly, we cannot observe the presence of Topo_C_Rpt in any bacterial species belonging to the phylum Firmicutes (Table 1).

The Topo_C_ZnRpt with four cysteines for Zn^2+^ coordination is referred to as zf-C4_Topoisom (PF01396) in the Pfam database. Most of the bacterial species that have Topo_C_ZnRpt are from the phyla of Proteobacteria and Firmicutes (Table 1 and Figure S2). Topo_C_Rpt is preferred over Topo_C_ZnRpt in Actinobacteria. Furthermore, it can be noted that 210 archaea species have Topo_C_ZnRpt, but no archaea species is listed for Topo_C_Rpt (Figure S1).

#### 2.1.2. Consensus Sequence for Topo_C_ZnRpt and Topo_C_Rpt

**Figure 2:**
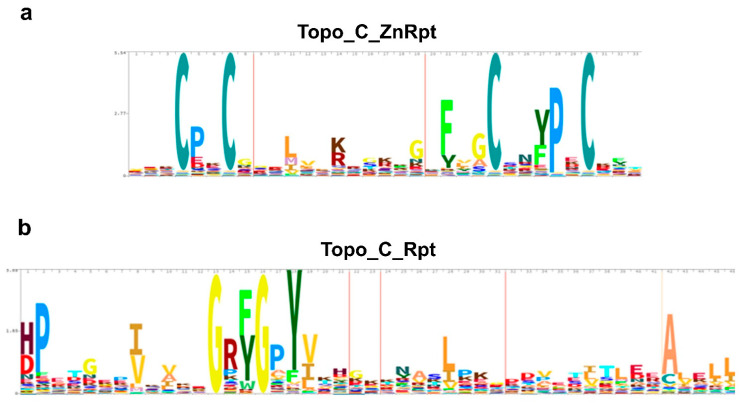
HMM logos for bacterial topoisomerase I C-terminal repeats. (**a**) Topo_C_ZnRpt (Pfam01396 with 14,012 sequences); (**b**) Topo_C_Rpt (Pfam13368 with 14,232 sequences).

The HMM Logo [25] for Topo_C_ZnRpt and Topo_C_Rpt as presented in the Pfam database for zf-C4_Topoisom (PF01396, 14,012 sequences) and Toprim_C_rpt (PF13368, 14,232 sequences) are shown in Figure 2. For Topo_C_ZnRpt, the first two cysteines for Zn^2+^ coordination are separated by two residues while the third and fourth cysteines are further apart. The residue that is two residues before the third cysteine is usually an aromatic residue that contributes one DNA-binding site, Figure 2a. The residue that is two residues after the third cysteine is also usually aromatic and contributes to the second DNA-binding site. These aromatic residues interact with two consecutive nucleotides of DNA with their side chains forming π–π stacking with the bases of the nucleotides as evidenced in the EcTOP1 structure in complex with DNA oligonucleotides [17]. The last two C-terminal domains D8 and D9 of EcTOP1 are Topo_C_ZnRpt homologs that have lost their zinc binding cysteines and are called zinc ribbon-like domains [16,26], or Topo_Zn_Ribbon (PF08272) in the Pfam database. However, their DNA-binding modes seem to be preserved, as shown in the structure of EcTOP1 with ssDNA bound to the C-terminal domains [17].

For Topo_C_Rpt, the signature GR (F/Y) GPY sequence is critical for DNA binding. The sidechains of the two conserved (F/Y) and Y residues contribute two DNA binding sites that have been observed in the co-crystal structures of *M. smegmatis* topoisomerase I with DNA oligonucleotides [21]. Besides these two aromatic residues that can interact with two consecutive nucleotides of substrate DNA through π–π stacking, the presence of an arginine residue in the sequence motif indicates potentially additional electrostatic interaction between the arginine to the phosphate groups of the DNA backbone. Although the interaction was not directly observed in the crystal structure, it may play roles during the recruitment of DNA substrate. The two glycines flanking R (F/Y) may provide some conformational flexibility for these two DNA-binding residues.

#### 2.1.3. Combinations of Topo_C_ZnRpt and Topo_C_Rpt Repeats in Individual Bacterial Topoisomerase I Sequences

EcTOP1 and MtbTOP1 are examples where only Topo_C_ZnRpt or Topo_C_Rpt is present in the individual bacterial topoisomerase I C-terminal region. Inspection of topoisomerase I protein sequences from a representative list of different classes of bacteria (Table S1) and the architectures listed in Pfam database for Topo_C_ZnRpt (Pfam01396) as well as Topo_C_Rpt (Pfam13368) revealed that these two types of C-terminal domains can appear together in different combinations in individual topoisomerase I sequences (Table 2). Partial gene duplication could potentially increase the number of repeats present in the 3′ region of the individual topoisomerase gene. The acquisition of additional C-terminal repeats could enhance the interaction between the type IA topoisomerase and nucleic acid substrates for greater efficiency in the topoisomerase physiological functions. Interestingly, when both types of C-terminal domains are present, the Topo_C_ZnRpt always follows D4 of the N-terminal toroid domains (Figure S3). The order of appearance of Topo_C_ZnRpt and Topo_C_Rpt in TopA of *Rickettsia bellii*, *Caulobacter crescentus,* and *Methylocapsa palsarum* (Table 2) are examples of such pattern illustrated in Figure S3.

### 2.2. Observation of New Types of C-Terminal Repeats in Fungal Topoisomerase III

**Figure 3:**
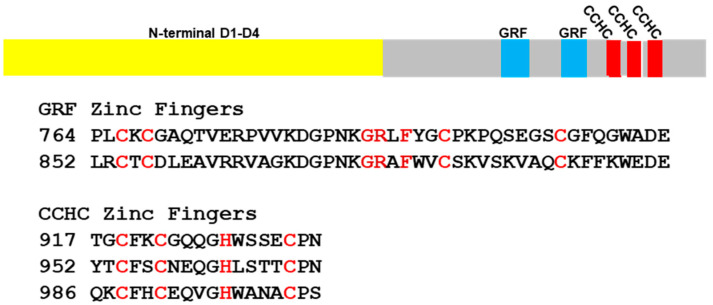
Position and alignment of zinc finger sequences found in topoisomerase III of *Puccinia graminis f.* sp. *tritici*. The cysteines and histidines for coordination of Zn^2+^ are colored in red. The conserved Gly, Arg, and Phe residues in the zf- GRF zinc fingers are colored in dark red.

While topoisomerase I encoded by the *topA* gene is often the only type IA topoisomerase present in a bacterial species, a subset of bacterial species has topoisomerase III present as an additional type IA topoisomerase that is mainly responsible for resolution of replication or recombination intermediates with its highly efficient decatenation activity and relatively weak relaxation activity [27,28]. *E. coli* topoisomerase III (EcTOP3) has also been shown to have a more robust RNA topoisomerase activity than EcTOP1 [29,30]. EcTOP3 encoded by the *topB* genes has a basic C-terminal region (~33 a.a.) [20] without any repeating units. The type IA topoisomerases present in eukaryotes are called topoisomerase III, and their N-terminal domains D1–D4 have greater homology to EcTOP3 than EcTOP1. Topoisomerase III (Top3) in higher eukaryotes have multiple zinc finger repeats in their C-terminal regions similar to the Topo_C_ZnRpt found in EcTOP1 [1]. However, fungal topoisomerase III from *Saccharomyces cerevisiae* [31] and *Schizosaccharomyces* *pombe* [32] has only a short basic region similar in length (~31 and 36 a.a.) to EcTOP3. We therefore tried to determine if repeat units for potential nucleic acid interactions can be found in type IA Top3 in other fungal species. Examination of fungal topoisomerase III sequences retrieved from the Uniprotein database showed that certain fungal topoisomerase IIIs do have repeats of zinc fingers classified in Pfam as zf-GRF (PF06839) or zf-CCHC (PF00098). The sequences of such zinc fingers found in topoisomerase III of *Puccinia graminis f.* sp. *tritici* (Uniprotein A0A5B0PD53) are shown in Figure 3 as an example. *P. graminis f.* sp. *tritici*, a devastating pathogen of crop plants, is the causal agent of wheat and barley stem rust [33].

#### 2.2.1. Distribution of Top3 C-Terminal Repeats in Fungal Phyla

The widely studied fungal species, *S. cerevisiae* and *S. pombe*, are members of the phylum Ascomycota. The OrthoDB listed 372 Top3 genes in 360 species in the phylum Ascomycota, with no zinc finger domains present in these Top3 genes. We examined the topoisomerase III protein sequences of 26 Ascomycota fungal species from various subphyla (Table S2). They all have a short basic C-terminal region (~30–40 a.a.) without any recognizable structural domains similar to Topo_C_Rpt, Topo_C_ZnRpt, or other zinc fingers. However, OrthoDB indicated the presence of zf-GRF and zf-CCHC zinc fingers in the 131 Top3 genes found in 130 species from the phylum Basidomycota. When topoisomerase III sequences from species in fungal phyla other than Ascomycota were examined, zf-GRF and zf-CCHC can be seen existing as repeated C-terminal domains. The fungal species that have zinc finger repeats in their topoisomerase III C-terminal domains include many members of the phylum Basidomycota (Table S2) that form the Dikarya subkingdom along with the phylum Ascomycota [34]. Agaricomycotina, Pucciniomycotina, Ustilaginomycotina, and Wallemiomycotina, the subphyla under Basidomycota [35], all have species with both zf-GRF and zf-CCHC zinc fingers in their topoisomerase III C-terminal region (Table S2). Some of the fungal species have more than one type IA Top3 present in the genome that may or may not contain the zinc fingers. For example, *Choanephora cucurbitarum* has two topoisomerase III with uniprotein IDs of A0A1C7NLX2 (548 residues, no zinc fingers) and A0A1C7N0U0 (749 residues, 2 zf-GRF). In addition to the Basidomycota phylum, zf-GRF can also be found in at least one the Top3 sequences for species from other fungal phyla [34] including Microsporida, Chytridiomycota, Cryptomycota, Blastocladiomycota, Zoopagomycota, and Mucoromycota. (Table S2). The zf-CCHC appears less frequently in the Top3 sequences examined than the zf-GRF and can be found mostly in Basidomycota. We did observe the presence of zf-CCHC in Top3 of *Coemansia reversa* in the phylum Zoopagomycota and *Rozella allomycis* in the phylum Cryptomycota.

#### 2.2.2. Combinations of zf-GRF and zf-CCHC Zinc Fingers in Fungal Species

Table 3 shows the different combinations of zf-GRF and zf-CCHC observed in the fungal Top3 sequences examined in this study. These zinc fingers vary in copy numbers in the Top3 C-terminal region. It can be noted that we did not find any fungal Top3 with only zf-CCHC and no zf-GRF in their C-terminal domains. Moreover, when both types of zinc fingers are present, the zf-GRF would follow the N-terminal domains and precede the zf-CCHC. This is similar to the preferred order of appearance of the Topo_C_ZnRpt before the Topo_C_Rpt observed in the bacterial topoisomerase I sequence that has both types of C-terminal repeats.

#### 2.2.3. Consensus Sequence for zf-GRF and zf-CCHC Zinc Fingers Found in Fungal Topoisomerase III

**Figure 4:**
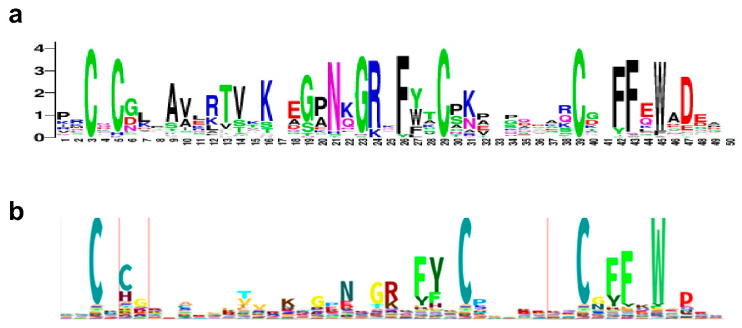
Comparison of consensus sequence for fungal Top3 zf-GRF from sequences of all zf-GRFs found in eukaryotes in the Pfam database. (**a**) Logo sequence for zf-GRF in fungal topoisomerase III (generated with WebLogo with 47 sequences from 27 species shown in Table S2). (**b**) HMM logo of all sequences in the Pfam database for zf-GRF (PF06839, sequences from 1341 species).

Figure 4 compares the consensus sequence of zf-GRF found in fungal topoisomerase III versus the Logo sequence available in Pfam database for zf-GRF present in all proteins in the database. The first two Zn^2+^-coordinating residues are separated by one residue. The third and the fourth Zn^2+^-coordinating Cys residues are separated by a variable number of residues. A significant portion of the zf-GRF sequences in Pfam has His as the second Zn^2+^-coordinating residue while all the fungal Top3 zf-GRF sequences use four Cys for Zn^2+^ coordination. Preference of NxGRxFY (Y = aromatic residue) in the region preceding the third Cys can be seen for the zf-GRF sequences in the fungal Top3 and Pfam database. The fungal Top3 zf-GRF sequences also have additional conserved residues in the region that follows the second Cys. A cluster of aromatic residues including two phenylalanines and one tryptophan after the last Cys is highly conserved across zf-GRF domains.

**Figure 5:**
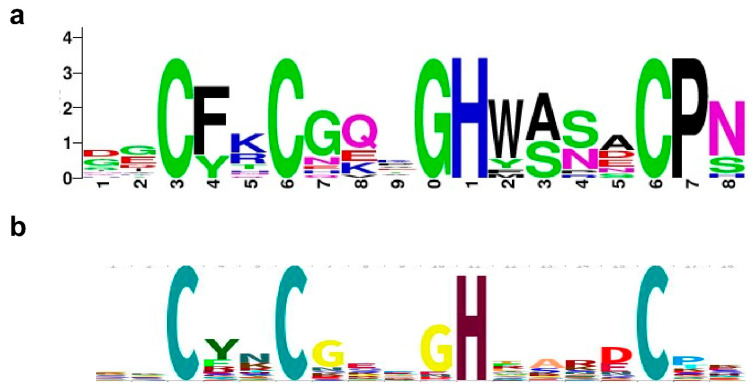
Comparison of consensus sequence for fungal Top3 zf-CCHC from sequences of all zf-CCHC found in eukaryotes in the Pfam database. (**a**) Logo sequence for zf-CCHC in fungal topoisomerase III (from 23 sequences found in 13 species shown in Table S2). (**b**) HMM logo of all sequences in the Pfam database for zf-CCHC (PF00098, sequences from 1680 species).

The consensus sequence of the fungal Top3 zf-CCHC (Figure 5) has the two glycines that are at the two ends of the loop connecting the second cysteine and histidine for Zn^2+^ coordination. Interestingly, there is a preference for an aromatic residue that follows the first Cys and His, as well as a proline that follows the fourth cysteine. A basic/polar residue is favored before the second cysteine and at the first position between the two glycines.

#### 2.2.4. Predicted Structures and Nucleic Acid Interactions for zf-GRF Domains of *Puccinia graminis f.* sp. *tritici* Topoisomerase III

**Figure 6:**
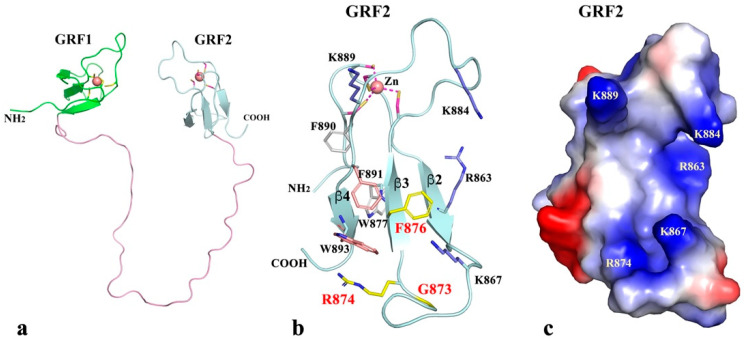
Predicted structures of zf-GRF repeats in the C-terminal region of *P. graminis f.* sp. *tritici* topoisomerase III. (**a**) Predicted structures for the two zf-GRF domains (GRF1 and GRF2) connected with a 40 residue linker. (**b**) A ribbon diagram of GRF2 domain. Besides the four cysteines that form the Zn^2+^-binding site, other key residues (including the signature GRxF motif labeled in red) that may contribute to domain folding and DNA-binding are drawn in stick format for highlighting. (**c**) Electrostatic surface potential representation of GRF2.

The structures of two zf-GRF domains in *Puccinia graminis f.* sp. *tritici* topoisomerase III have been predicted as described in Methods. The modeling of individual zf-GRF domains seemingly followed the three available zf-GRF structures, *Xenopus laevie* Apex2 C-terminal zf-GRF [36], human N^6^-methyladenosine N-terminal zf-GRF [37], and human NEIL3 C-terminal tandem zf-GRF domains [38]. The two zf-GRF domains (GRF1 and GRF2) are connected by a 40 residue long linker (Figure 6a). Each of two individual zf-GRF domains are featured with an antiparallel 3-stranded β-sheet (Figure 6b). The three strands are labeled as β2, β3, and β4, respectively, for comparison to a typical 4-stranded Topo_C_ZnRpt domain [17]. One of the key potential DNA-binding residues of zf-GRF, the phenylalanine residue of the GRxF motif (F876 in GRF2), is in the middle of the β3 strand (Figure 6b) [17]. The residue F is highly conserved even though GR (G873 and R874 in GRF2) has relatively lower frequency for appearing in this subset of the zinc finger family (Figure 4b). Both zf-GRF and Topo_C_ZnRpt domains are 4C zinc fingers that are similar in sizes. One of the unique features of zf-GRF is the presence of aromatic residues on its β4-strand and its approximate such as F891 and W893 in GRF2 in the front of its β-sheet and F890 and W877 in the back of the β-sheet. To W893 the R874 from the GRxF motif adds a cation-π stacking. It is not clear if or how this cluster of aromatic residues in the zf-GRF domain may contribute to DNA binding. They may enhance the structural stability of the zf-GRF domain. It may also be related to the absence of the β1-strand that is found in the Topo_C_ZnRpt domain [17]. Additionally, several positively charged residues, some more conserved than others, help create a DNA-binding groove in the front of the twisted β-sheet (Figure 6c).

Although the structure and function of each of two individual zf-GRF domains can be predicted to a certain extent, their possible association is unknown, especially in the presence of a 40 residue long linker between them. The two human NEIL3 C-terminal zf-GRF domains are packed against each other with a short 3-residue linker [38]. The association of the two zf-GRF domains was believed to enhance DNA binding and the binding specificity [38]. In the prototypical Topo_C_ZnRpt-containing EcTOP1 structure, there are two interacting pairs (D5-D6 and D8-D9) [17]. These observations seemingly suggest that the small zf-GRF domains and Topo_C_ZnRpt domains tend to form a domain–domain association for the benefits of increased DNA-binding and binding specificity as well as an expanded regulation role [17,38].

#### 2.2.5. Predicted Structures and Nucleic Acid Interactions for zf-CCHC Domains of *Puccinia graminis f.* sp. *Tritici* Topoisomerase III

**Figure 7:**
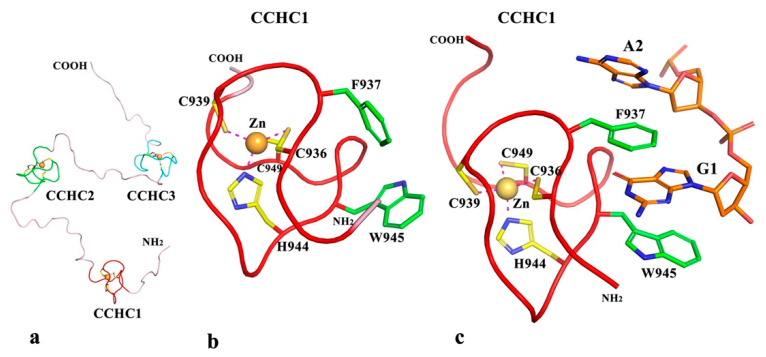
Predicted structures of zf-CCHC repeats in the C-terminal region of *P. graminis f.* sp. *tritici* topoisomerase III. (**a**) Predicted structures for the three zf-CCHC domains (CCHC1, CCHC2, and CCHC3) connected by flexible linkers. (**b**) A ribbon diagram of CCHC1. Besides the three cysteines and one histidine that form the Zn^2+^-binding site, two key residues (F937 and W945) that may contribute to DNA-binding are drawn in stick format. (**c**) A DNA-binding model of CCHC1 with a dinucleotide (GA).

The structures of the three zf-CCHC repeats in *Puccinia graminis f.* sp. *tritici* topoisomerase III have been predicted in a separate run as described in Methods. The structure of the typically 18 residue repeat, xxCxxCxxxxHxxxxCxx, is very conserved. The small domain has long been regarded as a single-stranded nucleic acid (RNA/DNA) binding zinc finger [39,40], but not exclusively [41]. Its binding modes to RNA/DNA have also been well characterized [42,43,44]. The modeling of the three zf-CCHC domains (CCHC1, CCHC2, and CCHC3) in *Puccinia graminis f.* sp. *tritici* topoisomerse III are straightforward (Figure 7a). They are linked by flexible loops, which are about 16 residues long each. The linkers between GRF2 and CCHC1 and the C-terminal tail after CCHC3 are also predicted to be flexible. In zf-CCHC domains from fungal topoisomerase III (Figure 5a), besides the highly conserved three cysteines and one histidine, the residue after the first cysteine is predominantly aromatic and the residue after the histidine is also mostly aromatic or at least hydrophobic. Although these two residues are separated by seven residues, their sidechains face each other in the three-dimensional structure of the small domain (Figure 7b). The two sidechains are positioned so that they can trap the base of a nucleotide (ssRNA/ssDNA) by means of a sandwich, forming at least one π-π stacking interaction or a stacked π-π structure (Figure 7c). Therefore, we predict that the three C-terminal zf-CCHC domains in this fungal topoisomerase III could potentially bind single-stranded RNA/DNA [42,43]. However, if these two key residues, especially the one after the first cysteine are non-aromatic, a zf-CCHC will unlikely be able to bind RNA/DNA. Thus, we can also predict that a large number of zf-CCHC repeats present in proteins do not bind RNA/DNA based on Figure 5b.

As shown in Figure 7c, one individual zf-CCHC domain can bind one nucleotide or two at the most. The question is if multiple zf-CCHC repeats are present in a polypeptide, such as the three zf-CCHC in the topoisomerase III of *Puccinia graminis f.* sp. *Tritici*, can these zf-CCHC repeats coordinate their efforts in RNA/DNA binding, especially when they are well separated in sequence? As we have discussed above about the association of zf-GRF and Topo_C_ZnRpt domains, the association of these small zinc-finger-containing domains can improve their RNA/DNA binding affinities and binding specificities. The association is supported by the pairing of zf-CCHC repeats when they are separated only by short linkers in some proteins [43,45]. However, multiple zf-CCHC repeats with short linkers can also exist in an extended form, like beads on a string [46]. In fungal topoisomerase III, it is unknown if the three zf-CCHC repeats can assemble into any forms of association in the presence of their long inter-repeat linkers.

## 3. Discussion

A major function of type IA topoisomerases is to relieve the topological stress from excess negative supercoiling. The diversity of their C-terminal DNA/RNA-binding auxiliary domains may represent a fine-tune of the catalytic function of individual type IA topoisomerases. It may also provide function-added roles for these enzymes. It is certainly informative to collect and analyze the available genomic data on the conserved sequence and arrangements of these C-terminal domains to provide a broad view of their appearance over the molecular evolution pathways. However, we are still in the early stage of elucidating the structure and function of these type IA topoisomerase C-terminal domains. While structural determination of individual full length type IA topoisomerase may be challenging due to the flexibility of the C-terminal domain linkers, use of cryo-EM in the future could potentially provide structures of complexes formed between the type IA topoisomerases, nucleic acid substrates, and their protein partners.

The Topo_C_ZnRpt domain in bacterial topoisomerase I could be converted into the Topo_Zn_Ribbon domain (zinc ribbon like domain) with loss of the Zn^2+^ coordinating cysteines. From sequence comparison and structural similarity, the zinc ribbon-like domains D8 and D9 of EcTOP1 are examples of bacterial topoisomerase I C-terminal domains that likely have arisen from loss of cysteines from Topo_C_ZnRpt domains (zinc ribbon domain) [16]. This conversion is certainly not exclusive to EcTOP1. According to the Pfam database, such zinc ribbon-like domain (PF08272) is repeated twice at the C-terminal end of 442 topoisomerase I sequences found in Gammaproteobacteria belonging to the phylum Proteobacteria. On the other hand, when the C-terminal region of bacterial topoisomerase I contains a mixture of Topo_C_ZnRpt and Topo_C_Rpt domains, Topo_C_Rpt domains are always located downstream of Topo_C_ZnRpt domains, similarly implying a possible evolutionary relationship between these two types of C-terminal domains, in which the Topo_C_ZnRpt domain is converted to the Topo_C_Rpt domain by losing Zn^2+^-binding site cysteines. The relative advantage of having Topo_C_ZnRpt or Topo_C_Rpt in the bacterial TopA C-terminal domains is not fully understood. The topoisomerase I proteins in the Alphaproteobacteria branch of Proteobacteria along with Actinobacteria and Bacteroidetes contain mainly Topo_C_Rpt while there are >1000 species in the phylum Firmicutes that have only Topo_C_ZnRpt domains (Table 1, Figure S2). The starkly contrasted distribution of two types of C-terminal domains in bacterial topoisomerase I needs to be further explored.

It is also interesting that when both zf-GRF and zf-CCHC are present in the fungal topoisomerase III sequences, the zf-GRF always precede the zf-CCHC. This is similar to the order of domain arrangement of the two zf-GRF and one zf-CCHC present in human topoisomerase III-alpha (Top3A) as shown in Figure S4. The two zf-GRF zinc fingers in human Top3A are preceded by two Topo_C_ZnRpt zinc fingers. Topoisomerase III-beta (Top3B) is the other type IA topoisomerase found in humans. Top3B has a cysteine-rich C-terminal region that could potentially form four C4-type zinc fingers (Figure S4). Except four expected zinc finger-forming cysteines in each domain, these four C4-type domains do not share further sequence similarity with either Topo_C_ZnRpt or zf-GRF domains.

In contrast to the close relationship between Topo_C_ZnRpt (zinc ribbon domain) and Topo_Zn _Ribbon (zinc ribbon-like domain) discussed above, there is no clear indication how zf-GRF and zf-CCHC domains are possibly related in terms of size, sequence, and fold at the molecular level. The distinctive adaption of these two types of zinc finger containing C-terminal domains in fungal topoisomerase III may arise from the different fungal life cycles. The Ascomycota and Basidiomycota phyla belong to the Dikarya subkingdom as they both possess two distinct nuclei during certain stages of their life cycles. However, the dikaryotic state of Ascomycota and Basidiomycota are expressed differently [34]. Ascomycota (sac fungi) form meiotic spores called ascospores that are enclosed in an ascus sac while Basidiomycota (club fungi) produce club-shaped spore-bearing end cells called basidia [34,47]. Clamp connections often maintain the long lasting dikaryotic state of many Basidiomycetes. It is possible that certain physiological processes in some of the Basidiomycota species may involve specific nucleic acid or protein interactions of the Top3 C-terminal domain zinc fingers. More detailed analysis of the variation in life cycle complexity, sexual reproduction, and genome maintenance of fungal species that possess topoisomerase III with C-terminal zinc fingers could provide clues on what selective advantage may lead to the acquisition and retention of these zinc-finger-containing repeats in Top3 of Basidiomycota and other fungal phyla, but not in Ascomycota.

To assist in the understanding of the distribution of these DNA/RNA-binding (or potential DNA/RNA-binding) C-terminal domains and further study, taxonomy common trees were generated with the NCBI tool for a representative subset of the bacterial (Figure S5) and fungal species (Figure S6) analyzed in this study. The numbers of the different C-terminal repeats found in the bacterial TopA and fungal Top3 in these species have been placed next to the species in the trees to illustrate the distribution among the phyla that were discussed. We did not present in this paper phylogenetic trees based on alignment of these type IA topoisomerases because such alignments would be dominated by the highly conserved N-terminal catalytic domains. The C-terminal domains have a low degree of homology, with a variable number of duplicated subdomain sequence motifs that most likely have come from horizontal gene transfer and gene duplication events. These events are known to cause disagreement between gene trees and species phylogeny [48].

The locating of zf-GRF and zf-CCHC types of repeats in the C-terminal region of fugal topoisomerase III has enriched our knowledge in the range of DNA/RNA-binding C-terminal domains of type IA topoisomerases. The knowledge may be extended further with increased interest in these nucleotide-binding domains for their roles in various DNA/RNA processing routes. It is noted that in addition to providing greater binding affinity and selectivity for DNA/RNA, these zinc-finger-containing domains could also potentially participate in protein–protein interactions. The Topo_C_ZnRpt of *E. coli* topoisomerase I has been shown to interact directly with RNA polymerase to facilitate removal of negative supercoils generated during rapid transcription and therefore prevent R-loop accumulation [22]. Zinc fingers are also present in the C-terminal domains of topoisomerase III of higher eukaryotes [1,3]. However, none of the structures of these C-terminal domains has been determined experimentally. It has been proposed that RMI1 and Top3A in the conserved BLM-Top3A-RMI1 (BTR) complex of *Arabidopsis* limit meiotic crossover formation through the interactions of the C-terminal domains of Top3A [49]. In germline of *Caenorhabditis elegans*, the single zinc finger C-terminal domain of topoisomerase III has been shown to cooperate with the RMI1 scaffold to promote stable association of the BTR complex to recombination intermediates [50]. With the systematic examination and preliminary characterization of zf-GRF and zf-CCHC types of C-terminal domains in fungal topoisomerase III presented in this study, identification of their interaction partners is likely to further elucidate the physiological functions of these type IA topoisomerases.

## 4. Materials and Methods

### 4.1. Sequence Database Search

Species were selected across different phyla and subphyla in the bacteria and fungi kingdom for representation of type IA topoisomerase sequence variation. Species analyzed include a diverse subset of bacterial species that contain TopA or fungal species that contain Top3 orthologues as listed in the Ortho DB v10, plus additional fungi species not listed in the Ortho DB. Protein sequence of TopA or Top3 in the species of interest was retrieved from the Uniprot database. The presence of C-terminal repeats was indicated by the Pfam database information in the Uniprot page for the topoisomerase. In some cases, additional C-terminal repeats of interest were identified through visual inspection for the presence of the conserved sequence motifs in the C-terminal region of the topoisomerase protein sequence.

### 4.2. Generation of Taxonomy Common Tree

Procedures provided in the NCBI Taxonomy database web site [51] were followed. The NCBI ID of 63 fungi species were retrieved from the Taxonomy database and entered into the NCBI web page for generating the Taxonomy Common Tree as described [51]. This process was repeated to generate the tree for 51 bacteria species.

### 4.3. Sequence Alignment

Alignments of all available sequences and HMM logo corresponding to Topo_C_ZnRpt and Topo_C_Rpt can be found in the Pfam database under PF01396 (zf-C4_Topoisom) and PF13368 (Toprim_C_rpt). Sequences corresponding to zf-GRF and zf-CCHC repeats identified in fungal Top3 listed in Table S2 were aligned using MUSCLE [52] for generating consensus sequence logos using WebLogo [53].

### 4.4. Structure Prediction and Model Building

The structure prediction of the two zf-GRF and three zf-CCHC domains of *Puccinia graminis f.* sp. *Tritici* topoisomerase III was performed by using AlphaFold2 [54] without providing any templates. Zinc ions were then manually added to those apparent metal binding sites of the predicted protein peptide only structures with the program COOT [55]. The resultant two zf-GRF domains and three zf-CCHC domains structures were subject to geometry minimization in Phenix [56]. The binding model of one zf-CCHC (CCHC1) to a dinucleotide (GA) was largely built based on the interaction of zf-CCHC1 of Lin28 to an oligonucleotide [57].

## 5. Conclusions

This study showed that type IA topoisomerases in both bacteria and fungi can have two distinct types of tandem C-terminal domains for potential interactions with nucleic acids and protein partners. The newly described distribution and combination of the Topo_C_Rpt and Topo_C_ZnRpt in bacterial TopA, as well as zf-GRF and zf-CCHC in fungal Top3 across different phyla pose interesting questions on how the observed arrangements of these C-terminal domains may be related to specific physiological functions of the type IA topoisomerases, and the biological adaptations of the species.

## Figures and Tables

**Table 1:** Number of species with Topo_C_ZnRpt or Topo_C_Rpt in individual bacterial phylum. Phyla with the greatest number of species listed in the Pfam database as having PF01396 (Topo_C_ZnRpt) or PF13368 (Topo_C_Rpt) are shown here.

Phylum ^1^	Topo_C_ZnRpt (PF01396)	Topo_C_Rpt (PF13368)
Actinobacteria	55	1003
Bacteroidetes	32	607
Firmicutes	1067	0
Proteobacteria	1679	1011

^1^ Examples of bacteria in other phyla that have either Topo_C_ZnRpt or Topo_C_Rpt in their topoisomerase I (TopA) sequence can be found in Table S1.

**Table 2:** Examples of species with the different combinations of repeated units of Topo_C_ZnRpt and Topo_C_Rpt observed in bacterial TopA. Numbers of Topo_C_ZnRpt and Topo_C_Rpt repeats found in topoisomerase I (TopA) of the species are shown here.

Species ^1^	Phylum	UniProt ID	Topo_C_ZnRpt	Topo_C_Rpt
*Acidobacterium capsulatum*	Acidobacteria	C1F6V0	5	0
*Mycobacterium avium*	Actinobacteria	X8B6F9	0	1
*Streptomyces inhibens*	Actinobacteria	A0A371PYR4	0	4
*Flavobacterium fontis*	Bacteroidetes	A0A1M5B513	0	2
*Caldilinea aerophila*	Chloroflexi	I0I048	0	3
*Lactobacillus plantarum*	Firmicutes	A0A0G9F8X8	2	0
*Staphylococcus aureus*	Firmicutes	Q2FZ32	3	0
*Caulobacter crescentus*	Proteobacteria	Q9A5J6	1	3
*Helicobacter pylori*	Proteobacteria	P55991	4	0
*Methylocapsa palsarum*	Proteobacteria	A0A1I4AKP7	1	4
*Rickettsia bellii*	Proteobacteria	Q1RIM1	1	2
*Thermotoga maritima*	Thermotoga	P46799	1	0

^1^ Additional examples of bacterial species with the different combinations of numbers of Topo_C_ZnRpt and Topo_C_Rpt repeat units can be found in Table S1.

**Table 3:** Examples of species with the different combinations of repeated units of zf-GRF and zf-CCHC observed in fungal topoisomerase III. Numbers of zf-GRF and zf-CCHC found in this individual topoisomerase III (Top3) are shown here.

Species ^1^	Phylum	UniProt ID	Zf-GRF	Zf-CCHC
*Candida auris*	Ascomycota	A0A0L0P6P7	0	0
*Wallemia ichthyophaga*	Basidiomycota	R9AS06	1	1
*Grifola frondosa*	Basidiomycota	A0A1C7M1I3	1	2
*Steccherinum ochraceum*	Basidiomycota	A0A4R0RRI7	1	3
*Ustilago maydis*	Basidiomycota	A0A0D1C790	2	2
*Puccinia graminis f.* sp. *tritici*	Basidiomycota	A0A5B0PD53	2	3
*Spizellomyces punctatus*	Chytridiomycota	A0A0L0HVJ1	1	0
*Rozella allomycis*	Cryptomycota	A0A075AT24	3	1 ^2^
*Rhizopus azygosporus*	Mucoromycota	A0A367JWR4	2	0
*Coemansia reversa*	Zoopagomycota	A0A2G5B3Y2	2	1

^1^ Additional examples of fungal species with the various combinations of numbers of zf-GRF and zf-CCHC repeat units shown here can be found in Table S2. ^2^ The zf-CCHC starting at residue 1190 of A0A075AT24 is not listed in the Pfam database.

## Data Availability

Not applicable.

## Supplementary Materials

The following supporting information can be downloaded at: https://www.mdpi.com/article/10.3390/ijms23158709/s1.
