# Supplementary material for: Microbial Type IA Topoisomerase C-Terminal Domain Sequence Motifs, Distribution and Combination
Source: Int J Mol Sci. 2022 Aug 5;23(15):8709. doi: 10.3390/ijms23158709 (PMC9369019; doi:10.3390/ijms23158709)
Supplement: Supplementary file 1 [file ijms-23-08709-s001.zip › Revised Supplementary Figures.pdf]

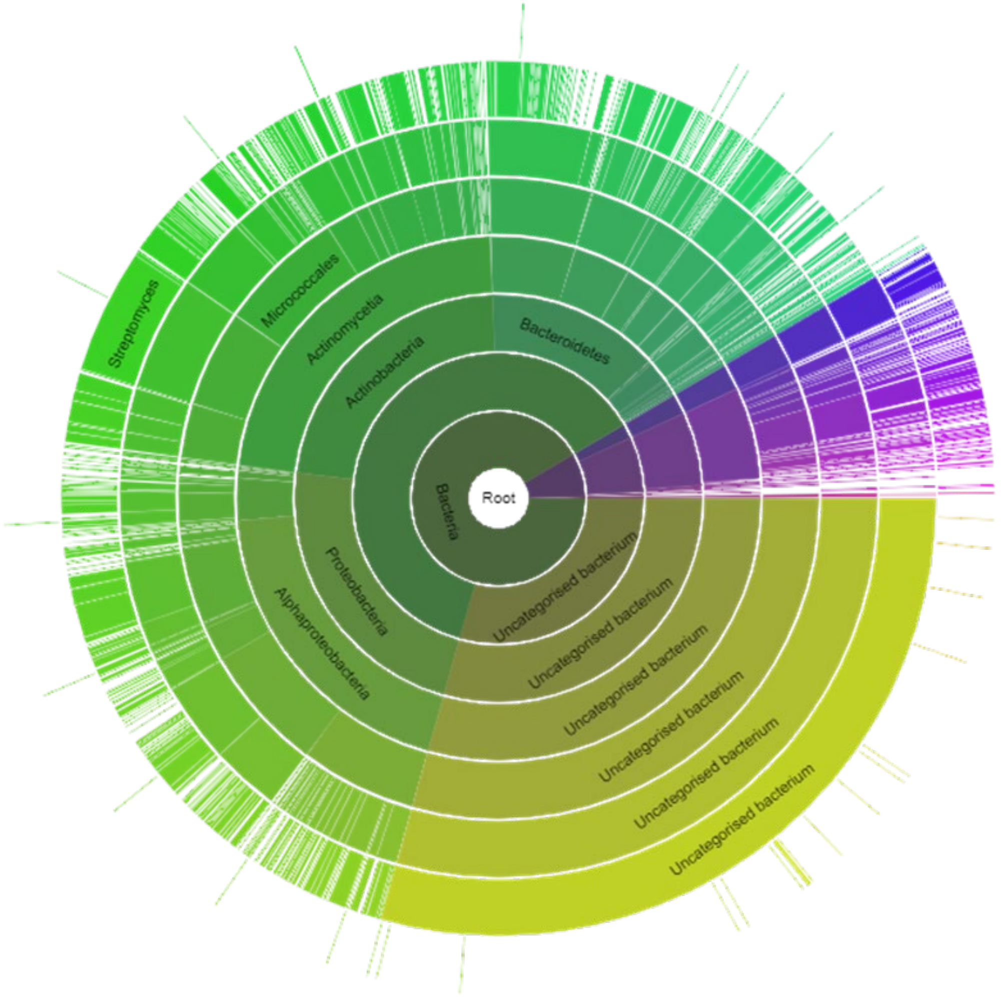


Figure S1. Sunburst illustration of species with Topo_C_Rpt. n=4235 (from http://pfam.xfam.org/family/Toprim_C_rpt#tabview=tab7). Color assignments: Green – Bacteria, Purple – Eukaryota.

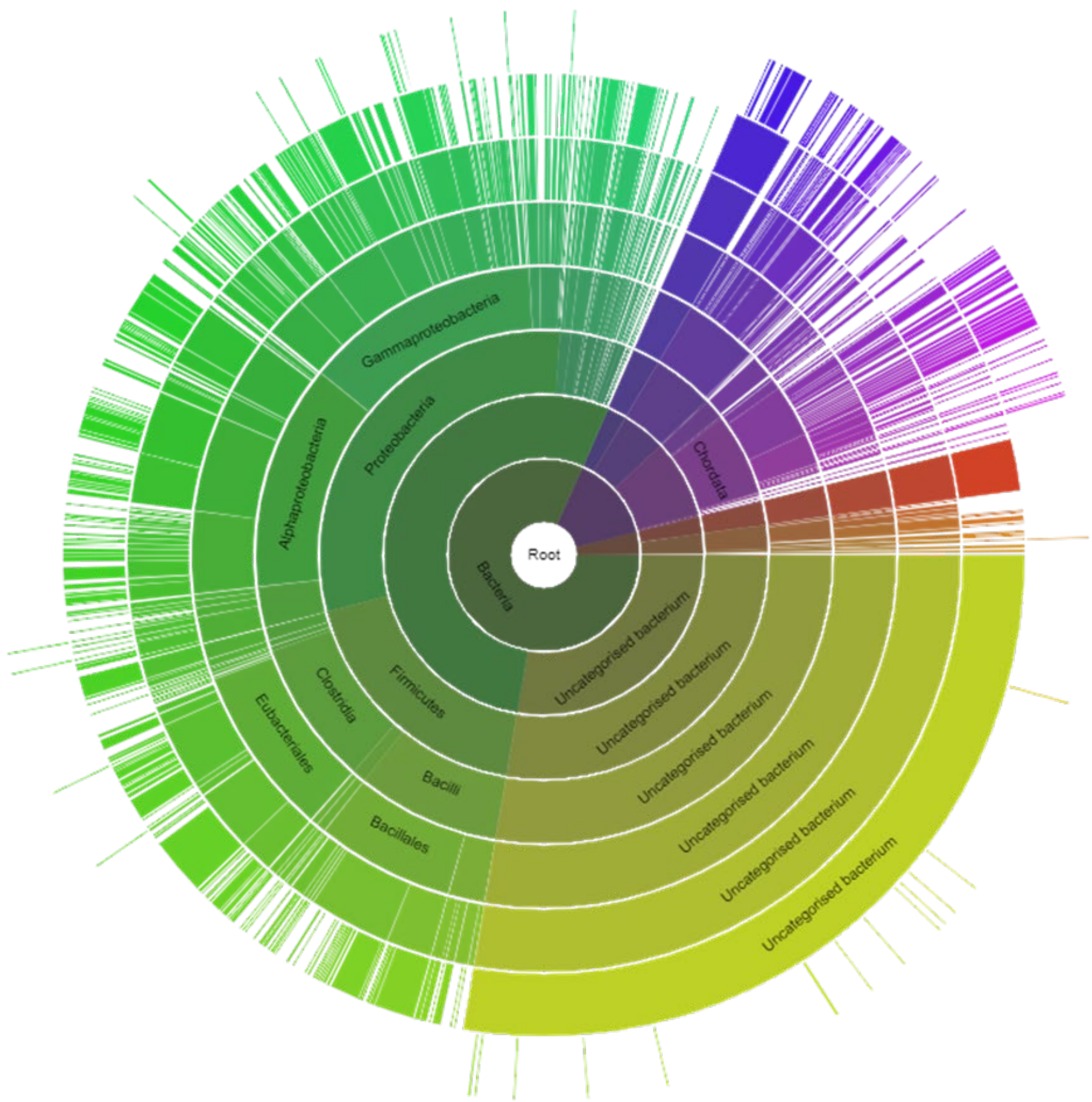


Figure S2. Sunburst illustration of species with Topo_C_ZnRpt. n=5401 (from http://pfam.xfam.org/family/zf-C4_Topoisom#tabview=tab7). Color assignments: Green – Bacteria, Purple – Eukaryota, Orange - Archea.

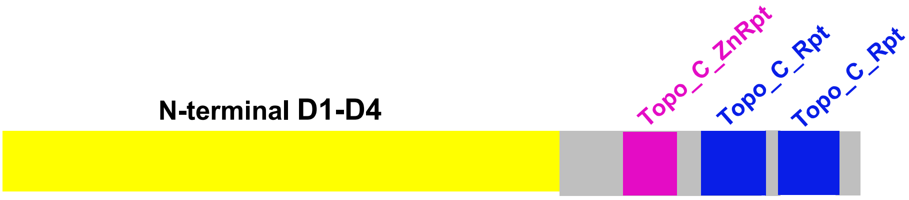


**TopA with one Topo_C_ZnRpt followed by two Topo_C_Rpt domains**
Examples of bacterial species: *Rickettsia bellii, Candidatus Phycorickettsia trachydisci*

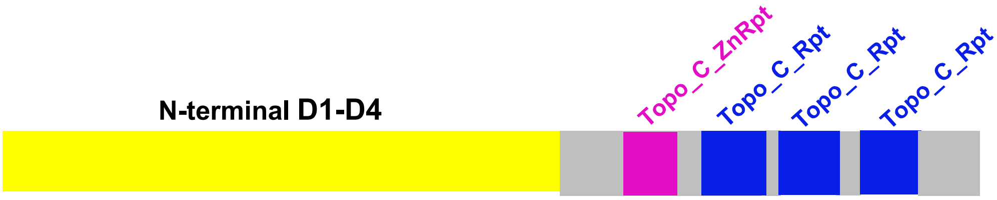


**TopA with one Topo_C_ZnRpt followed by three Topo_C_Rpt domains**
Examples of bacterial species: *Caulobacter crescentus, Gallionella capsiferriformans*

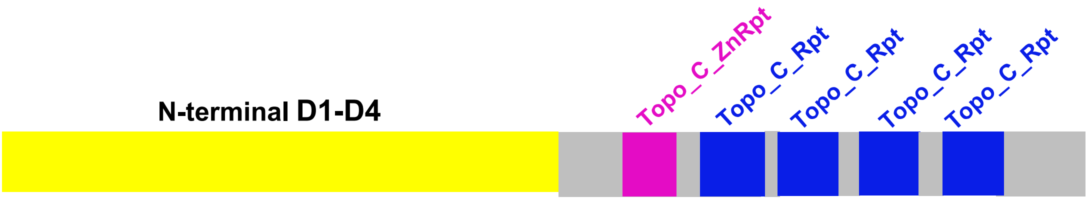


**TopA with one Topo_C_ZnRpt followed by four Topo_C_Rpt domains**
Examples of bacterial species: *Methylocapsa palsarum, Roseiarcus fermentans*

**Figure S3. Arrangements of Topo_C_ZnRpt and Topo_C_Rpt C-terminal domains in TopA from bacterial species found to have both types of C-terminal domains**

**Human Top3A**

**Topo_C_ZnRpt domains**
653 EPIRKCPQCNKDMVLKTKKN----------GGFYLSCMGFPECRSAVWLPDSVLEAS
700 RDSSVCPVCQPHPVYRLKLKFKRGSLPPTMPLEFVCCIGGCDDTLREILDLRFS

**Zf-GRF domains**
807 ESNSVTCNCGQEAVLLTVRKEGPNRGRQFFKCNGGSCNFFLWADSPNP
891 SGSGTSCLCSQPSVTRTVQKDGPNKGRQFHTCAKPREQQCGFFQWVDENTAP

**CCHC domain**
979 KKPRKCSLCHQPGHTRPFCPQNR

**Human Top3B**

**C4-type zinc fingers**
615 AATGKPLSRCGKCHRFMKYIQ----------AKPSRLHCSHCDETYTLPQNGTIKLYKELRCPLDD
679 GSRGKSYPLCPYCYNHPPFRD-----------MKKGMGCNECTHPSCQH
717 SLSMLGIGQCVECESGVLVLDPTS-------GPKWKVACNKCNVVAHCFENA
762 HRVRVSADTCSVCEAALLDVDFNKAKSPLPGDETQHMGCVFCDPVFQELVELKHAASCHPMH

**RGG motif**
824 RGGPGRRQGRGRGRARRPPGKPNPRRPK

Figure S4. C-terminal domains in human type IA topoisomerases. The RGG motif in Top3B is important for RNA binding.

| Taxon | Topo_C_ZnRpt | Topo_C_Rpt |
|---|---|---|
| Bacteria | | |
| Cyanobacteria | | |
| **Gloeocapsa sp. PCC 7428** | 0 | 3 |
| **Synechococcus elongatus PCC 7942 = FACHB-805** | 0 | 4 |
| Bacteroidetes | | |
| **Rhodocytophaga rosea** | 0 | 3 |
| Flavobacteriaceae | | |
| **Formosa sp. Hel1_31_208** | 0 | 2 |
| **Spongiivirga citrea** | 0 | 2 |
| **Aurantibacter aestuarii** | 0 | 2 |
| **Mangrovimonas yunxiaonensis** | 0 | 2 |
| **Flavobacterium fontis** | 0 | 2 |
| **Winogradskyella sp. PG-2** | 0 | 2 |
| **Rhodothermus marinus DSM 4252** | 0 | 3 |
| Chlamydia | | |
| **Chlamydia pneumoniae** | 3 | 0 |
| **Chlamydia trachomatis D/UW-3/CX** | 3 | 0 |
| Actinobacteria | | |
| **Berryella intestinalis** | 3 | 0 |
| Actinomycetia | | |
| Streptomyces | | |
| **Streptomyces inhibens** | 0 | 4 |
| **Streptomyces coelicolor A3(2)** | 0 | 4 |
| Mycobacterium | | |
| **Mycobacterium avium subsp. avium 2285 (R)** | 0 | 1 |
| **Mycobacterium tuberculosis H37Rv** | 0 | 4 |
| Proteobacteria | | |
| Alphaproteobacteria | | |
| **Candidatus Cytomitobacter primus** | 1 | 2 |
| Rhodospirillales | | |
| **Caenispirillum bisanense** | 1 | 3 |
| **Roseomonas cervicalis ATCC 49957** | 1 | 3 |
| Rhodobacterales | | |
| **Albimonas donghaensis** | 1 | 3 |
| Roseobacteraceae | | |
| **Sulfitobacter mediterraneus** | 1 | 3 |
| **Rubellimicrobium mesophilum DSM 19309** | 1 | 3 |
| Hyphomicrobiales | | |
| Beijerinckiaceae | | |
| **Beijerinckia sp. 28-YEA-48** | 1 | 4 |
| **Methylocapsa palsarum** | 1 | 4 |
| **Roseiarcus fermentans** | 1 | 4 |
| Methylocystaceae | | |
| **Methylosinus sp. R-45379** | 1 | 4 |
| **Methylocystis sp. SC2** | 1 | 4 |
| **Aquamicrobium defluvii** | 1 | 3 |
| **Agrobacterium fabrum str. C58** | 1 | 3 |
| **Rickettsia bellii RML369-C** | 1 | 2 |
| **Zymomonas mobilis subsp. mobilis ZM4 = ATCC 31821** | 1 | 3 |
| **Caulobacter vibrioides CB15** | 1 | 3 |
| **Helicobacter pylori 26695** | 3 | 0 |
| Gammaproteobacteria | | |
| **Xylella fastidiosa 9a5c** | 0 | 3 |
| **Escherichia coli K-12** | 3 | 0 |
| Firmicutes | | |
| Bacilli | | |
| Lactobacillales | | |
| **Lactiplantibacillus plantarum** | 2 | 0 |
| **Enterococcus faecalis** | 3 | 0 |
| **Streptococcus pneumoniae** | 3 | 0 |
| Bacillales | | |
| **Listeria monocytogenes serotype 4b str. LL195** | 3 | 0 |
| Bacillaceae | | |
| **Halalkalibacterium halodurans C-125** | 3 | 0 |
| **Bacillus subtilis subsp. subtilis str. 168** | 3 | 0 |
| **Staphylococcus aureus subsp. aureus NCTC 8325** | 3 | 0 |
| Eubacteriales | | |
| **Sulfobacillus acidophilus DSM 10332** | 3 | 0 |
| **Clostridioides difficile 630** | 3 | 0 |
| **Caldilinea aerophila DSM 14535 = NBRC 104270** | 0 | 3 |
| **Thermotoga maritima MSB8** | 1 | 0 |
| **Deinococcus radiodurans R1** | 0 | 4 |
| **Borreliella burgdorferi B31** | 0 | 1 |
| **Aquifex aeolicus VF5** | 0 | 0 |

Figure S5. Taxonomy common tree of select bacterial species to illustrate the distribution of Topo_C_ZnRpt and Topo_C_Rpt repeats in bacterial topoisomerase I. The number of the repeats found in the TopA C-terminal region are indicated next to the species.

| Taxon | zf-GRF | zf-CCHC |
|---|---|---|
| Fungi | | |
| Rozella allomycis CSF55 | 3 | 1 |
| Microsporidia | | |
| Nematocida parisii ERTm1 | 2 | 0 |
| Enterocytozoonidae | | |
| Enterospora canceri | 1 | 0 |
| Hepatospora eriocheir | 0 | 0 |
| Enterocytozoon hepatopenaei | 1 | 0 |
| Nosema bombycis CQ1 | 2 | 0 |
| Ascomycota | | |
| Leotiomycetes | | |
| Pseudogymnoascus verrucosus | 0 | 0 |
| Blumeria hordei DH14 | 0 | 0 |
| Botrytis cinerea B05.10 | 0 | 0 |
| Saccharomycetales | | |
| [Candida] auris | 0 | 0 |
| Cyberlindnera jadinii NRRL Y-1542 | 0 | 0 |
| Saccharomyces cerevisiae S288C | 0 | 0 |
| Scheffersomyces stipitis CBS 6054 | 0 | 0 |
| Protomyces lactucae-debilis | 0 | 0 |
| Hypocreales | | |
| Metarhizium rileyi | 0 | 0 |
| Fusarium oxysporum f. sp. cubense | 0 | 0 |
| Dothideomycetes | | |
| Lasiodiplodia theobromae | 0 | 0 |
| Bipolaris victoriae FI3 | 0 | 0 |
| Zymoseptoria tritici IPO323 | 0 | 0 |
| Aspergillaceae | | |
| Penicillium brasilianum | 0 | 0 |
| Monascus purpureus | 0 | 0 |
| Aspergillus | | |
| Aspergillus niger CBS 513.88 | 0 | 0 |
| Aspergillus flavus NRRL3357 | 0 | 0 |
| Aspergillus fumigatus Af293 | 0 | 0 |
| Xylona heveae TC161 | 0 | 0 |
| Pneumocystis | | |
| Pneumocystis jirovecii RU7 | 0 | 0 |
| Pneumocystis murina B123 | 0 | 0 |
| Orbiliaceae | | |
| Dactylellina haptotyla CBS 200.50 | 0 | 0 |
| Orbilia oligospora ATCC 24927 | 0 | 0 |
| Pezizales | | |
| Pyronema omphalodes CBS 100304 | 0 | 0 |
| Tuber melanosporum Mel28 | 0 | 0 |
| Schizosaccharomyces pombe 972h- | 0 | 0 |
| Chytridiomycota | | |
| Neocallimastix californiae | 0 | 0 |
| Chytridiomycetes | | |
| Batrachochytrium dendrobatidis JEL423 | 2 | 0 |
| Spizellomyces punctatus DAOM BR117 | 1 | 0 |
| Gonapodya prolifera JEL478 | 2 | 0 |
| Mucoromycota | | |
| Mucorales | | |
| Choanephora cucurbitarum | 2 | 0 |
| Rhizopus azygosporus | 2 | 0 |
| Rhizophagus irregularis DAOM 181602=DAOM 197198 | 0 | 0 |
| Basidiomycota | | |
| Dacrymycetaceae | | |
| Dacryopinax primogenitus | 2 | 0 |
| Calocera viscosa TUFC12733 | 0 | 0 |
| Malassezia | | |
| Malassezia vespertilionis | 0 | 0 |
| Malassezia sympodialis ATCC 42132 | 1 | 1 |
| Malassezia globosa CBS 7966 | 0 | 0 |
| Malassezia restricta CBS 7877 | 0 | 0 |
| Tremellomycetes | | |
| Tremellales | | |
| Saitozyma podzolica | 2 | 1 |
| Cryptococcus neoformans var. neoformans JEC21 | 2 | 1 |
| Apiotrichum porosum | 1 | 0 |
| Agaricomycetes | | |
| Hypsizygus marmoreus | 2 | 0 |
| Polyporales | | |
| Steccherinum ochraceum | 1 | 3 |
| Grifola frondosa | 1 | 2 |
| Microbotryomycetes | | |
| Leucosporidium creatinivorum | 2 | 2 |
| Sporidiobolaceae | | |
| Sporidiobolus salmonicolor | 1 | 0 |
| Rhodotorula graminis WP1 | 2 | 3 |
| Wallemia ichthyophaga EXF-994 | 1 | 1 |
| Mixia osmundae IAM 14324 | 2 | 0 |
| Ustilaginaceae | | |
| Sporisorium reilianum SRZ2 | 2 | 2 |
| Ustilago maydis 521 | 2 | 2 |
| Puccinia graminis f. sp. tritici | 2 | 3 |
| Zoopagomycota | | |
| Basidiobolus meristosporus CBS 931.73 | 0 | 0 |
| Conidiobolus coronatus NRRL 28638 | 0 | 0 |
| Coemansia reversa NRRL 1564 | 2 | 1 |
| Allomyces macrogynus ATCC 38327 | 1 | 0 |

Figure S6. Taxonomy common tree of select fungi species to illustrate the distribution of zf-GRF and zf-CCHC repeats in fungal topoisomerase III. The number of the repeats found in the Top3 C-terminal region are indicated next to the species.
